# Diabetes knowledge and associated factors in adolescents and young adults with type 1 diabetes in Ouagadougou (Burkina Faso)

**DOI:** 10.1186/s12902-023-01469-1

**Published:** 2023-09-30

**Authors:** Daniel Zemba, Yempabou Sagna, Solo Traore, Lassane Zoungrana, D. Patricia Somé, S. Aimée Kissou, Oumar Guira, Téné Marceline Yaméogo

**Affiliations:** 1 Service de médecine interne, CHU Yalgado Ouédraogo, Ouagadougou, Burkina Faso; 2 Unité de Formation et de Recherche en Sciences de la Santé, Université Joseph KI-ZERBO, Ouagadougou, Burkina Faso; 3 Institut Supérieur des Sciences de la Santé, Université Nazi BONI, Bobo Dioulasso, Burkina Faso; 4 Service de Médecine Interne, CHU Sourô Sanou, Bobo Dioulasso, Burkina Faso; 5 Service de Pédiatrie, CHU Sourô Sanou, Bobo Dioulasso, Burkina Faso

**Keywords:** Type 1 diabetes knowledge, Adolescent, Burkina Faso

## Abstract

**Background:**

Type 1 diabetes (T1D) in youth is becoming a public health problem in Sub-Saharan Africa, including Burkina Faso. However, little is known about the level of knowledge of these patients on T1D. This study aimed to evaluate the knowledge of diabetes in adolescents and young adults about the disease, and identify the factors associated in Burkina Faso.

**Methods:**

A cross-sectional survey was conducted from April to June 2020 among youth with T1D, aged 10 to 30 years, and regularly followed in the internal medicine department of Yalgado Ouedraogo University Hospital of Ouagadougou, Burkina Faso. Data were collected using the French AJD (Aide aux Jeunes Diabétiques) validated diabetes knowledge and skills (DKS) questionnaire designed to test participants’ accuracy in knowledge about six different themes of T1D, as generalities of diabetes, hypoglycemia and hyperglycemia, management of insulin treatment, and self-monitoring blood glucose (SMBG). DKS level was determined by calculating the scores, and univariate and multivariate logistic regression were used to explore factors influencing DKS scores. This level was classified as insufficient or low ≤ 25/50, regular 26–39/50, and adequate or good ≥ 40/50.

**Results:**

Sixty-three participants with a mean age of 19.05 years and a sex ratio (W/M) of 1.17 were included in our study. The mean HbA1c level was 9.79%, and 43 (68.23%) patients had an insufficient DKS level. The mean global DKS score of correct answers was 23.63/50. The percentage of correct answers was respectively 50% for the item “generalities of diabetes”, 32.4% for the item “hypoglycemia and hyperglycemia”, 67.72% for the item “diet”, 37.34% for the item “management of insulin treatment” and 44.97% for the item “SMBG”. In univariate analysis, a better patient DKS level was associated with university education and long duration of diabetes care follow-up (> 10 years, p < 0.05). Only increasing age remain associated with a better knowledge score (p < 0.05) in multivariate analysis.

**Conclusion:**

This study is an important first step in identifying areas for intervention efforts about therapeutic education for youth with type I diabetes in Burkina Faso.

**Supplementary Information:**

The online version contains supplementary material available at 10.1186/s12902-023-01469-1.

## Background

Type 1 diabetes (T1D) is the most frequently type of diabetes in youth, characterized by chronic immune-mediated destruction of pancreatic β-cells, usually leading to absolute insulin deficiency [1]. Achieving target glucose levels assessed through monitoring blood glucose levels and glycated hemoglobin is essential to minimize the detrimental effects of hypoglycemia and hyperglycemia [2]. Most of youth with T1D from sub-Saharan African do not meet recommended glycemic targets [3].

The Diabetes Control and Complications Trial and its follow-up study, the Epidemiology of Diabetes Interventions and Complications study, confirmed that an improvement in long-term glucose control by intensified insulin therapy and extensive support and education, can reduce the incidence of complications and delay the progression of existing complications in T1D, in adolescents and adults [4].

Since the implementation of the Life for a Child (LFAC) program [5] in Burkina Faso, prevalence of people with T1D < 25 years rose from 0.14/100,000 in 2013 from 1.36/100,000 by 2021 [6].

In Burkina Faso, because of limited access to insulin and high prices, most of young people with TID benefit from LFAC at the internal medicine department (the main referral center for diabetes in the country) of Yalgado Ouedraogo Teaching Hospital (CHU YO) in Ouagadougou. This program commenced an intervention to provide care for all young people < 26 years of age with diabetes in Burkina Faso in 2013, donating insulin and glucose-monitoring supplies. The internal medicine department of the CHU YO also usually follows people with T1D over the age of 25 years who previously benefit from the LFAC program. These young people followed in the CHU YO have been seen as routine visit every three or four months. They come from all regions of the country except western and south-western regions where there is another hospital with the LFAC support. As of April 1, 2020, out of 143 patients aged 25 and under were followed in Burkina Faso throughout LFAC support, 108 (75.52%) were followed at CHUYO center.

The insulin donated by LFAC was mostly premixed 70/30 insulin formulation, so patients were mainly given fixed doses. All participants routinely test their blood glucose at home two times per day but only those on multiple daily injection insulin therapy were encouraged to adjust their insulin dose at home with meals, exercise, or blood glucose readings. For those on fixed doses, they were encouraged to adjust only in case of recurrent hypoglycemia. Glycemic control in children and adolescents with T1D in this center is very poor [7].

As part of insulin treatment, achieving good glycemic target requires a comprehensive diabetes education [8]. Therapeutic patient education (TPE) allows patients to improve their knowledge and skills both on their chronic disorder and their treatment [9]. In sub Saharan Africa, TPE on diabetes is limited among children with diabetes [10]. Consultation times are short, resulting in little or no time for patient education [11].

All young people followed in the CHU YO center benefit sometimes from therapeutic education for diabetes monitoring, which had remained mainly individual during their routine visits, with a few group sessions on diet since the end of 2018. Educative tools used were those of the French educative program published as “Les Cahiers de l’AJD” (Aide aux Jeunes Diabétiques, help to young diabetics) [12].

In Africa, several studies assessed diabetes knowledge in adults’ patients with type 2 and type 1 diabetes [13–18], showing the insufficient level of knowledge (≤ 50% of correct answers through submitted questionnaires). But very few addressed to young people with T1D [19, 20]. In Burkina Faso there is only one study on this topic for type 2 diabetes (T2D) [21] but none for young people with T1D, and the TPE program conducted in the CHUYO center was still not evaluated.

To fill this gap, we aimed to assess T1D knowledge in adolescents and young adults about the disease, and identify the factors associated in the CHUYO center.

## Methods

This was a single center cross-sectional survey study conducted from April 1st to June 30th, 2020. The study population included adolescents and young adults with T1D, aged 10 to 30 years, and regularly followed in the internal medicine department of CHUYO for at least one year. As diabetes autoantibodies and C-peptide assays are not available in Burkina Faso, T1D was diagnosed according to standard World Health Organization criteria [22]. Such criteria included age, presentation, the abrupt onset of symptomatic hyperglycemia, need for insulin replacement therapy from diagnosis, and no suggestion of T2D or another type of diabetes being responsible.

People with another type of diabetes, and those with mental ill-health were excluded. The study was performed during regular outpatient consultations or hospitalizations in this center. All consecutive patients who met the inclusion criteria and seen during the study period have been included. Among them, 6 were aged < 10 years and 9 had less than 1 year history of diabetes and were excluded to the study. All included patients were diagnosed before the age of 25years, as they were all previously followed up in our Life For A Child cohort patients.

We used the AJD (Aide aux Jeunes Diabétiques) diabetes knowledge and skills (DKS) adapted French questionnaire for children and adolescents with T1D [23]. This questionnaire is composed of 50 true-false questions designed to test participants’ accuracy in knowledge about six different themes of T1D: 6 questions on the generalities of diabetes, 12 on hypoglycemia and hyperglycemia, 9 on diet, 17 on the management of insulin treatment and 6 about self-monitoring blood glucose (SMBG). The process of developing the questionnaire was designed based on 310 true-false questions elaborated from a program aimed at initial education of T1D, “les Cahiers de l’AJD”, with the participation of 1576 10–20 years old T1D adolescents, 466 parents, 33 pediatric centers and eight AJD diabetes camps. Answers were analyzed by linkage studies (Ward’s minimum variance method) to guide the Educative Committee in selecting one-third of the questions; the 105 remaining questions were retested in a single series by 564 T1D adolescents, while a test-retest was also performed by 77 adolescents or parents, to select 67 questions, before a last test by 200 adolescents for the final selection of the 50 questions we used [23].

The AJD DKS questionnaire was completed by the patients. In case of French language obstacle, the questionnaire was administered by the interviewer (doctor) with translation of the questionnaire into the two most national languages speaking (“Dioula” and “Mooré”). It was the same interviewer for all patients. To minimize alteration of the questions, we first tested them to some patients who spoke both French and one or the other of the 2 translation languages to ensure that the understanding was the same.

Each correct answer was scored as “1” and each incorrect answer scored as “0”. We associated the choice answer option “I don’t know” to optimize the rate of correct answers. For the analysis we made a two-stage model where the first part was the variables that predict thinking that patient know the answer (yes = 1 and no = 0) to assess only the response of “I do not know”. The second part of the model was the variables that predict the answer they give conditional on thinking that they know (correct answer = 1, incorrect answer = 0).

The score of DKS was evaluated by the number of correct answers to the AJD questionnaire. The level of diabetes knowledge was empirically classified according to total number of correct answers, insufficient or low ≤ 25/50 (≤ 50% of correct answers), regular 26–39/50 (51–79%), and adequate or good ≥ 40/50 (≥ 80%).

For each patient, the consulting physician completed a file to collect the following data: date of first insulin injection, number of daily injections, times of injections (breakfast, lunch, afternoon snack, dinner, and bedtime) and types of insulin (fast-acting, long-lasting, and premixed).

Descriptive analyses are presented as mean (± SD) for quantitative variables and as frequencies and percentage for qualitative variables. Correlation analyses between DKS total score and factors included in the analysis were performed using Chi-square test (or Fisher’s test if appropriate), with a significance level of 0.05. In the multivariate analysis, we included variables that are known to affect knowledge from literature even if they may not have been significant in the univariate analysis, such are sex, age, schooling, living or not with parents, follow up duration and HbA1c. Analyses were made with the IBM SPSS Statistics 26 software, 2018.

Information collected about patients was treated confidentially.

Informed written consent from the youth included, or from their parents or guardians for young people under 18, was obtained before their inclusion in the study. We also sought assent from the children aged 10–17 yrs. The patient’s refusal to participate in this study in no way prevented his treatment and follow-up in the center. The National ethic committee (Comité d’Ethique pour la Recherche en Santé) provided the approval for the study with the registration No 2020-8-146.

## Results

Sixty-three participants with a mean age of 19.05 years and a sex ratio (F/M) of 1.17 were included in our study. Female represented 53.97% of participants.

Thirty-eight (60.32%) participants had primary or intermediate school level of education and 8 (12.7%) had high school level. Fifty (78.24%) participants lived in urban areas and 40 (63.5%) lived with both their two parents. More than 20 km separated the home from the monitoring center for 33.33% of youth.

The mean age at the discovery of diabetes was 14.88 years and the circumstance of discovery was ketoacidosis in 50% of cases. The mean duration of diabetes was 3.47 ± 2.51 years. In 68.3% of cases, the duration of diabetes was less than 5years.

Fifty-three participants (84.12%) had fixed doses of insulin. After the initial hospitalization, 26 (41.27%) of patients were re-hospitalized at least once for a total of 31 acute complications, including 9 cases (34.61%) of severe hypoglycemia and 22 cases (84.61%) of ketoacidosis.

The mean HbA1c level at the last contact was 9.79% with an average insulin dose of 0.78 IU/kg/day.

Regarding the TPE, 32 patients (67%), felt well to very well educated.

### DKS level

The mean global score of correct answers was 23.63/50. Forty-three (68.23%) patients had an insufficient level of knowledge on their illness and their treatment.

About the sections of the DKS questionnaire, the percentage of correct answers of each theme was respectively 50% for the item “generalities of diabetes”, 32.4% for the item “hypoglycemia and hyperglycemia”, 67.72% for the item “diet”, 37.34% for the item “management of insulin treatment” and 44.97% for the item “SMBG” (Table 1).

**Table 1:** Adolescents and young adults DKS score

	Number of questions	DKS score [range], (% of correct answers)
Global score	50	23,63 [23–41] (47.26)
Score per item		
Generalities of diabetes	6	3.49 [1–5] (50)
Hypo and hyperglycemia	12	4.41 [1–10] (32.4)
Diet	9	3.33 [1–7] (67.72)
Management of insulin treatment	17	7.28 [2–13] (37.34)
Self-monitoring blood glucose	6	2.31 [0–6] (44.97)

DKS, Diabetes Knowledge, and skills

In univariate analysis (Table 2), a better DKS score was associated with university education and long duration (> 10 years) of follow-up (p < 0.05). There was no significant difference in score according to sex, age, urban or rural origin, parental situation, feeling of being well educated or having a good glycemic control, DKA episodes and age at T1D diagnosis (p > 0.05).

In multivariate analysis (Table 3), only age was associated with a better knowledge score (p < 0.05). There was no difference in score between patients according to their glycemic control, sex, and family status.

**Table 2:** Associations between the youth DKS score of correct answers and clinical, HbA1c and socio demographic characteristics in univariate analysis

Variable	n	DKS score of correct answers, %	p
Sex			
Girls	29	47.44 ± 12.43	0.9
Boys	34	47.11 ± 12.6	
Age class, years			
[10 à 19]	25	45.84 ± 12.5	0.08
≥ 20	38	51.48 ± 12.45	
Residency			
Urban	59	48.36 ± 12.43	0.6
Rural	4	45.5 ± 12.62	
Schooling			
Not schooled	17	41.73 ± 12.64	
Primary or secondary school	38	48.91 ± 12.43	0.06
High school degree	8	58.85 ± 12.56	0.005
Family situation			
Lives with both parents	40	47.93 ± 12.43	
Lives with one parent	4	51.5 ± 13.25	0.3
Lives with a guardian	19	48.82 ± 12.64	0.7
Follow up duration, years			
[1–4]	38	48.06 ± 12.43	
[5–9]	20	47 ± 12.42	0.7
≥ 10	5	64.5 ± 12.48	0.008
Feeling to have a good DKS	36	50.12 ± 12.69	0.2
Feeling to have a good glycemic control	25	46.45 ± 12.68	0.7
HbA1c < 8%	8	48.5 ± 12.77	0.7
At least 1 DKA episode	18	44 ± 12.36	0.3
Age at diabetes diagnosis, years			
<10	7	47.5 ± 12.83	
[10–19]	37	48.25 ± 12.71	
≥ 20	19	56.45 ± 12.59	0.06

DKA, Diabetes Keto Acidosis; DKS, Diabetes Knowledge, and skills; HbA1c, glycated hemoglobin

**Table 3:** Associations between the youth DKS score of correct answers and clinical, HbA1c and socio-demographic characteristics in multivariate analysis

Variable	Relative SD [95% CI]	p
Sex	0.03 [-0.06; 0.12]	0.555
Age	0.02 [0.01; 0.03]	0.004
Living with parents	-0.05 [-0.15; 0.05]	0.326
Schooling	0.06 [-0.04; 0.16]	0.245
Follow up duration	0.08 [-0.06; 0.21]	0.253
HbA1c	-0.01 [-0.03; 0.01]	0.485

CI, confidence interval; HbA1c, glycated hemoglobin; SD, Standard Deviation

## Discussion

With a rate of 47.26% of correct answers, the level of knowledge of adolescents and young adults with diabetes was insufficient. This level of knowledge is also found insufficient in another sub-Saharan African country like Malawi [19]. Our rate is near to the same of 48.2% found in Saoudi Arabia (63.8% of the child used insulin two times a day) [24] but less than the 86% found in one study in the USA [25]. These studies in Malawi, Saoudi Arabia and USA used the Michigan Diabetes Knowledge Test [26], which looked at general knowledge on diabetes, complications and self-care practices. We used the same questionnaire as in France where the level of knowledge is 75% [27]. The better level of DKS in these countries could be explained as their clinics usually offer comprehensive diabetes education by certified diabetes educators and a registered dietitian [23, 25, 27].

Nevertheless, in multivariate analysis, we only found age as the factor associated with the DKS. Age is also described as an associated factor in other studies [19, 27]. The study in Malawi shown association between age and DKS but regarding that 63% of participants had at least 5 years duration of diabetes, it is possible that duration powered the role of age. Other social and demographic factors associated are urban residency [28] and socioeconomic status [25]. Considering that 87.3% of our sample not having a university education and lived in urban areas, the lack of knowledge could be explained by the lack of TPE and the low level of school education.

There is a significant relationship between education level and diabetes knowledge [19, 27, 28], as we found in our study.

Glycemic control was not correlated with knowledge of diabetes as it is shown in another study in Brazil [29] where they also found a significant correlations between HbA1c and resilience, anxiety and depression. However, studies in the USA and France found that better diabetes knowledge is associated with glycemic control [25, 27], which may suggest the involvement of others factors like socioeconomic status or illness perception for example. Moreover, we think if our study was powered, some factors that have been associated with DKS score like glycemic control would have turned out significant. It was described in South Africa that adolescents with at-risk glycemic control believed that T1D is difficult to manage, leading to a largely negative perception of the disease [30].

Among the subsections of the DKS questionnaire, there was an average level of knowledge for the item “generalities” and a weak level for the other items (hypoglycemia, hyperglycemia, insulin treatment, SMBG). These findings are in agreement with the findings in Malawi [19], which contributes to poor glycemic control. As most of our patients had fixed doses of insulin and were not encouraged to adjust their insulin dose at home with meals, exercise, or blood glucose readings, this would impact their knowledge in these subsections of the DKS. It is recommended that SMBG is essential for diabetes management for all children and adolescents with diabetes and an inability to articulate symptoms of hypoglycemia impose to set higher HbA1c goal [2]. Moreover, management of insulin must be supported by comprehensive education, whatever insulin regimen is chosen [31].

There was a good knowledge for the item “diet”, which item is already found to be associated with a better diabetes knowledge in youth with T1D and their caregivers [25]. This good knowledge in our study could also be explained by the group’s education sessions focused on diet we started in the clinic since 2018.

Some limitations of this study may be sources of biases. First our study was limited to the single center of the CHU YO, not considering patients from the western and south-western regions of the country. The translation of the questionnaire from French into the national languages ​​"Dioula” and “Mooré” for its administration to young people who did not understand French language could be the source of misunderstandings. At last, in our analysis we did not do a distinction on the origin of young people between rural and urban areas.

Nevertheless, the CHU YO being the center receiving most people T1D in the country, the results of this study could be able to guide the initiation of therapeutic education programs for the benefit of all people with T1D.

## Conclusion

The DKS was insufficient. This research is an important first step in identifying a significant need to develop relevant TPE on diabetes for children, adolescents, and their parents within Burkina Faso to help them manage the condition hence averting long term complications.

### Electronic supplementary material

Below is the link to the electronic supplementary material.

Supplementary Material 1

## Data Availability

The datasets used and analyzed during the current study are available from the corresponding author upon reasonable request.

## Abbreviations

AJD: Aide aux Jeunes Diabétiques
CHU YO: Yalgado Ouedraogo Teaching Hospital
DKS: diabetes knowledge and skills
HbA1c: glycated hemoglobin
LFAC: Life for a Child
T1D: type 1 diabetes
T2D: type 2 diabetes
TPE: therapeutic patient education
F/M: female/male
