# Supplementary material for: Diabetes knowledge and associated factors in adolescents and young adults with type 1 diabetes in Ouagadougou (Burkina Faso)
Source: BMC Endocr Disord. 2023 Sep 30;23:210. doi: 10.1186/s12902-023-01469-1 (PMC10544174; doi:10.1186/s12902-023-01469-1)
Supplement: Supplementary file 1 — Supplementary Material 1 [file 12902_2023_1469_MOESM1_ESM.docx]

Supplementary file : Final adapted version of the AJD questionnaire of knowledge with 50 True-False questions. (*from Martin D, Dossier C, Godot C et al. Questionnaire de connaissance de l’AJD pour les enfants et les adolescents ayant un diabète de type 1. Educ Thérapeutique Patient - Ther Patient Educ 2016; 8: 10106.)*

| **N°** | **Questions** | **True** | **False** | **I don’t know** |
| --- | --- | --- | --- | --- |
|  | I look for acetone in the urine (or in the blood) when the blood glucose level is above 2.5g/l (13 mmol/l) |  |  |  |
|  | A blood glucose level of 0.85 g/l (4.6 mmol/l) is hypoglycemia |  |  |  |
|  | There is no danger in injecting glucagon |  |  |  |
|  | Glycated hemoglobin should be measured at least 3 times a year |  |  |  |
|  | It is better to do the morning insulin injection in the arms and/or stomach |  |  |  |
|  | A bowl of tea and a slice of bread make a balanced breakfast |  |  |  |
|  | It is recommended to play sports when you have acetone in the blood or in the urine |  |  |  |
|  | Diabetes is caused by insulin deficiency |  |  |  |
|  | I add a supplement of rapid acting insulin when I have hyperglycemia with glucose and acetone in the urine (or blood) |  |  |  |
|  | A glycated hemoglobin of 10% is a good result (the normal value being less than 6%) |  |  |  |
|  | The signs of ketoacidosis are nausea (feeling sick), vomiting, stomachache, rapid breathing |  |  |  |
|  | If I do physical exercise that was not planned, I eat an extra snack |  |  |  |
|  | I can adjust my insulin doses with just one blood glucose test per day |  |  |  |
|  | It is recommended to make insulin injections in areas of lipodystrophy |  |  |  |
|  | Evening rapid-acting insulin exerts its effect at the end of the night |  |  |  |
|  | After the injection of Glucagon, consciousness usually returns within about ten minutes. |  |  |  |
|  | If I urinate often, I think I lack insulin |  |  |  |
|  | The examination of the fundus allows the search for retinopathy (disease of the retina) |  |  |  |
|  | If yesterday I experienced a hypoglycemia without any cause identified, today I reduce the dose of insulin |  |  |  |
|  | At mealtimes I need more insulin than between meals |  |  |  |
|  | Orange, tangerine, and grapefruit are fruits higher in glucose than grapes and cherries |  |  |  |
|  | A headache on waking or nightmares may be manifestations of hypoglycemia |  |  |  |
|  | The amount of food you eat has no effect on blood glucose level |  |  |  |
|  | The action of insulin is accelerated by muscular exercise |  |  |  |
|  | If I have hyperglycemia at the time of the injection, I increase the dose of rapid-acting insulin |  |  |  |
|  | Regular insulin (HUMULIN R, ACTRAPID) acts for 4 to 6 hours* |  |  |  |
|  | If I have done a lot of sport at the end of the afternoon, I reduce the dose of evening rapid-acting (before dinner) |  |  |  |
|  | If I have acetone in my urine and normal blood glucose level, I take a rapid-acting insulin supplement |  |  |  |
|  | If I inject with a fold of skin, it's to prick into the muscle |  |  |  |
|  | Fever, strong emotions, and stress most often lower blood glucose level |  |  |  |
|  | During hypoglycemia, I take either bread or sugar |  |  |  |
|  | Sugar, starches, and fruits all contain carbohydrates |  |  |  |
|  | Midday analyzes allow the morning rapid-acting insulin dose to be adjusted |  |  |  |
|  | In case of hyperglycemia with acetone (in the blood or urine) I do analyzes at least every 4 hours until the disappearance of the acetone |  |  |  |
|  | A blood glucose level of 1.50 g/l (8 mmol/l) before a meal is normal |  |  |  |
|  | I eat vegetables at will |  |  |  |
|  | You can keep a vial or cartridge of insulin that has been opened for about 3 months outside the refrigerator. |  |  |  |
|  | Meats and cheeses should be consumed in moderation |  |  |  |
|  | If I weigh 40 kg, I take 1 sugar in case of hypoglycemia |  |  |  |
|  | Insulin-dependent diabetes is caused by eating too much sugar |  |  |  |
|  | The search for albumin in the urine (microalbuminuria) makes it possible to detect kidney disease (nephropathy) |  |  |  |
|  | To do a blood glucose measurement, I usually prick the middle of the finger |  |  |  |
|  | I inject HUMULIN R or ACTRAPID 1 hour before meals* |  |  |  |
|  | Hyperglycemia makes you gain weight |  |  |  |
|  | The more I balance my diabetes, the more I delay complications |  |  |  |
|  | Meat and fish are foods high in glucose |  |  |  |
|  | I lower my insulin dose if yesterday my results were too high |  |  |  |
|  | A can of soda contains the equivalent of 7 squares of sugar* |  |  |  |
|  | When I can't eat (sickness, vomiting), insulin treatment can be stopped |  |  |  |
|  | If you have diabetes, you cannot be vaccinated. |  |  |  |

**adapted questions*
